# Radar-Based Road Surface Classification Using Range-Fast Fourier Transform Learning Models

**DOI:** 10.3390/s25185697

**Published:** 2025-09-12

**Authors:** Hyunji Lee, Jiyun Kim, Kwangin Ko, Hak Han, Minkyo Youm

**Affiliations:** Convergence Reasearch Center for Disaster & Safety, Advanced Institute of Convergence Technology (AICT), Suwon 16229, Republic of Korea; leehyunji94@snu.ac.kr (H.L.); kjy615@snu.ac.kr (J.K.); kwang4372@snu.ac.kr (K.K.); hockey@snu.ac.kr (H.H.)

**Keywords:** mmWave radar, Frequency-Modulated Continuous Wave (FMCW), road condition monitoring, Range-FFT, machine learning, deep learning

## Abstract

Traffic accidents caused by black ice have become a serious public safety concern due to their high fatality rates and the limitations of conventional detection systems under low visibility. Millimeter-wave (mmWave) radar, capable of operating reliably in adverse weather and lighting conditions, offers a promising alternative for road surface monitoring. In this study, six representative road surface conditions—dry, wet, thin-ice, ice, snow, and sludge—were experimentally implemented on asphalt and concrete specimens using a temperature and humidity-controlled chamber. mmWave radar data were repeatedly collected to analyze the temporal variations in reflected signals. The acquired signals were transformed into range-based spectra using Range-Fast Fourier Transform (Range-FFT) and converted into statistical features and graphical representations. These features were used to train and evaluate classification models, including Extreme Gradient Boost (XGBoost), Light Gradient-Boosting Machine (LightGBM), Convolutional Neural Networks (CNN), and Vision Transformer (ViT). While machine learning models performed well under dry and wet conditions, their accuracy declined in hazardous states. Both CNN and ViT demonstrated superior performance across all conditions, with CNN showing consistent stability and ViT exhibiting competitive accuracy with enhanced global pattern-recognition capabilities. Comprehensive robustness evaluation under various noise and blur conditions revealed distinct characteristics of each model architecture. This study demonstrates the feasibility of mmWave radar for reliable road surface condition recognition and suggests potential for improvement through multimodal sensor fusion and time-series analysis.

## 1. Introduction

In recent years, traffic accidents caused by black ice during winter have emerged as a serious societal concern. In South Korea, a total of 3944 black ice-related accidents occurred between 2019 and 2023, resulting in 95 fatalities—a fatality rate of 2.4%, approximately 1.7 times higher than that of general traffic accidents [1]. A similar issue has also been reported in the United States. According to the Federal Highway Administration (FHWA), around 24% of weather-related vehicle crashes occur on snowy, slushy, or icy pavement annually, leading to over 1300 deaths and approximately 116,800 injuries. During active snowfall or sleet, approximately 900 fatalities and 76,000 injuries are recorded each year [2]. This high level of risk is primarily due to the visual indistinguishability of black ice and the challenge it poses for timely driver recognition.

Such accidents tend to concentrate during early morning hours in winter, when low temperatures and reduced visibility significantly impair the performance of conventional sensor-based detection systems. Optical sensors suffer drastic performance degradation in nighttime and adverse weather conditions, while infrared and thermal cameras can only measure surface temperature, making it difficult to distinguish between actual freezing and simple cooling. Acoustic sensors also face challenges due to small reflection differences and high sensitivity to environmental noise and other interferences [3,4,5,6].

These limitations have led to increasing demand for alternative technologies capable of reliably monitoring road surface conditions regardless of lighting or weather [7,8,9,10]. Research utilizing millimeter-wave (mmWave) radar has gained momentum [11], and recent systems have shown improved accuracy in recognizing diverse materials and surface conditions [12,13,14]. By operating at high frequency bands, mmWave radar can provide robust reflected signals even in snow, fog, or low-light environments, allowing for quantitative assessment of surface conditions and making it a promising alternative for ice detection [15,16,17].

Recent studies have used polarized mmWave radar operating in the 87.5–90.5 GHz range to classify surface conditions (dry, wet, icy), while other approaches combined 24 GHz mmWave radar with statistical features and machine learning to classify 12 typical road surface types and conditions (e.g., asphalt, gravel, wet, icy) [18,19,20]. Some researchers developed models based on meteorological statistics and imagery to identify snowy and icy surfaces [21]. Other studies applied Range-FFT analysis to train models that classify road objects or enhance detection speed and accuracy by integrating multiple deep learning techniques [22]. Techniques to differentiate between wet and dry roads using reflectivity strength or threshold-based freezing detection have also been proposed using 24–79 GHz radar [23,24,25,26,27]. Additionally, the use of range-Doppler maps has expanded detection capabilities beyond basic range and velocity, enabling classification of moving objects versus static surfaces and improving classification accuracy through deep learning [28], and reliably detecting moving objects under various weather conditions [29,30,31,32].

More recently, multimodal approaches have emerged as promising alternatives for road surface classification. Multimodal transformer models that integrate time-series data from multiple sensors have demonstrated enhanced feature integration capabilities and improved classification accuracy [33]. These approaches leverage the complementary strengths of different sensor modalities, such as radar, LiDAR, and optical sensors, to achieve more robust and accurate road surface recognition. However, such multimodal systems often require complex sensor fusion algorithms and may not be suitable for cost-sensitive applications where single-sensor solutions are preferred. Despite growing efforts to analyze reflection characteristics across various materials, movement-based experiments remain limited due to practical constraints [34].

While previous research has proposed road surface-recognition methods based on mmWave radar and Range-FFT analysis, most experiments have been confined to simplified laboratory conditions and basic surface states (e.g., dry, wet, ice). Although these approaches are valuable for technical validation, few studies have systematically evaluated the temporal behavior of reflected signals under dynamically changing surface conditions. In particular, there is a lack of research that investigates time-series changes in Range-FFT signals across dry, wet, thin-ice, ice, snow, and sludge conditions through repeated experiments to analyze reproducibility and classification performance. Previous studies were confined to simplified laboratory conditions and basic surface states (dry, wet, ice), lacking comprehensive evaluation of hazardous conditions such as thin-ice, snow, and sludge. Most existing research failed to systematically evaluate the temporal behavior of reflected signals under dynamically changing surface conditions, particularly the transition phases between different states. Conventional machine learning models showed significant accuracy decline when classifying dangerous surface conditions, limiting their practical applicability for safety-critical applications. Few studies investigated time-series changes in Range-FFT signals across multiple surface conditions through repeated experiments to analyze reproducibility and classification performance.

Despite the growing body of research on mmWave radar-based road surface recognition, several critical limitations remain unaddressed. Most existing studies have been confined to simplified laboratory conditions and basic surface states (e.g., dry, wet, ice), lacking comprehensive evaluation of hazardous conditions such as thin-ice, snow, and sludge that pose the greatest safety risks. Previous research has failed to systematically evaluate the temporal behavior of reflected signals under dynamically changing surface conditions, particularly the transition phases between different states that are crucial for real-time monitoring. Conventional machine learning models have shown significant accuracy decline when classifying dangerous surface conditions, limiting their practical applicability for safety-critical applications. Few studies have investigated time-series changes in Range-FFT signals across multiple surface conditions through repeated experiments to analyze reproducibility and classification performance. Current approaches primarily rely on basic statistical features (mean, max, std, median, mode) without leveraging more sophisticated time-frequency analysis techniques that could improve classification accuracy. While traditional machine learning methods have been applied, recent advances in ensemble learning, stacking models, and deep learning architectures have not been fully explored for road surface-classification tasks [35].

To address these research gaps, this study makes several key contributions to the field of road surface condition monitoring:

- Comprehensive surface condition analysis: Six representative road surface states (dry, wet, thin-ice, ice, snow, and sludge) were systematically implemented on both asphalt and concrete specimens using a controlled temperature and humidity chamber, enabling thorough analysis of hazardous conditions.
- Temporal signal behavior investigation: Radar data were collected at fixed time intervals to quantitatively observe transitions in surface conditions, providing insights into the dynamic behavior of reflected signals during state changes.
- Dual-approach feature extraction: The collected data were processed using Range-FFT to generate range-based spectra, from which both statistical features and image representations were extracted, enabling comprehensive classification strategies.
- Comprehensive model evaluation: These features were applied to conventional machine learning algorithms (XGBoost, LightGBM, Random Forest, SVM), CNN-based deep learning models, and Vision Transformer (ViT) to comprehensively evaluate classification performance across different surface conditions and material types.
- Advanced deep learning comparison: The study provides detailed performance analysis and comparison between CNN and ViT models, demonstrating their distinct characteristics and capabilities in road surface condition classification.
- Robustness evaluation framework: Comprehensive robustness testing under various noise and blur conditions was conducted for both CNN and ViT models, providing insights into their degradation patterns and practical applicability in challenging environments.

The rest of this paper is organized as follows: Section 2 describes the materials and methods, including mmWave radar background, experimental setup, data processing techniques, and model architectures (CNN and ViT). Section 3 presents the experimental results and performance analysis of different classification models, including comprehensive robustness evaluation under various noise and blur conditions. Section 4 provides a comprehensive discussion of the findings, including detailed comparison between CNN and ViT models, analysis of their distinct characteristics, comparison with existing literature, uncertainty analysis, and limitations. Finally, Section 5 concludes the study and outlines future research directions.

This comprehensive approach enables reliable road surface condition recognition and suggests potential for improvement through multimodal sensor fusion and time-series analysis in real-world applications.

## 2. Materials and Methods

### 2.1. Millimeter-Wave Radar Background

Millimeter-wave (mmWave) radar is a high-frequency sensing technology that operates in the 30–300 GHz band. Due to its short wavelength, it offers high spatial resolution, making it suitable for a wide range of applications such as communication networks [36,37], autonomous vehicles [38,39], human sensing [40], and structural health monitoring [41,42]. The core components of mmWave radar include signal modulation, transmission and reception, and signal processing. Among various modulation schemes, Frequency-Modulated Continuous Wave (FMCW) is most commonly employed.

#### 2.1.1. FMCW (Frequency-Modulated Continuous Wave)

**Figure 1:**
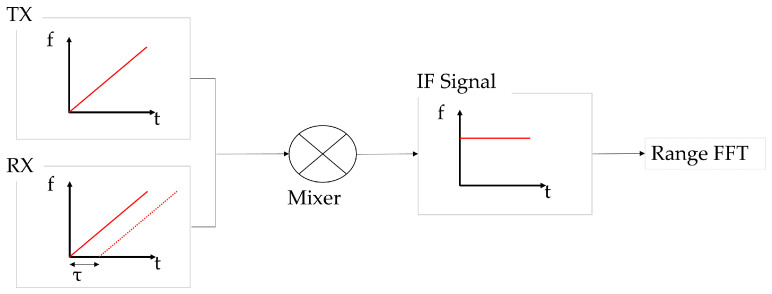
Graphical representation of the IF signal.

FMCW radar transmits a continuous electromagnetic wave (chirp) whose frequency varies linearly over time and receives its reflection from the target to estimate parameters such as range, velocity, and angle of arrival [43]. As illustrated in Figure 1, by measuring the frequency difference—known as the beat frequency—between the transmitted and received signals, accurate distance estimation becomes feasible. The FMCW method offers advantages such as compactness and low power consumption, which make it highly suitable for real-time sensing systems like the one used in this study [44].

The transmitted signal of the FMCW radar can be expressed as [45]:

(1) $$s_{\mathrm{tx}}\left(t\right)=A\cos\left(2\pi \left(f_{0}t+\frac{K}{2}t^{2}\right)\right)$$

where $f_{0}$ is the starting frequency, *K* is the chirp rate (frequency slope), and *A* is the signal amplitude.

The received signal reflected from the target with a time delay $\tau =\frac{2R}{c}$ due to the round-trip distance *R* is given by [45]:

(2) $$s_{\mathrm{rx}}\left(t\right)=A\cos\left(2\pi \left(f_{0}\left(t-\tau \right)+\frac{K}{2}{\left(t-\tau \right)}^{2}\right)\right)$$

By mixing the transmitted and received signals, the intermediate frequency (beat signal) is obtained as [45]:

(3) $$s_{\mathrm{beat}}\left(t\right)=\cos\left(2\pi f_{b}t+\phi \right)$$

Since the beat frequency $f_{b}$ is linearly proportional to the target range *R*, the distance can be estimated using [45]:

(4) $$R=\frac{c\cdot f_{b}}{2K}$$

#### 2.1.2. Raw Data Processing

The raw data collected from the FMCW radar consists of complex-valued samples arranged in a multidimensional array composed of frames, transmit antennas, receive antennas, chirps, and ADC samples. Although initially stored as a one-dimensional array, the data is reshaped during preprocessing into a five-dimensional structure, as shown in Equation (5) [46]:

(5) $$\mathrm{RawData}\in C^{N_{f}\times N_{\mathrm{chirp}}\times N_{\mathrm{tx}}\times N_{\mathrm{rx}}\times N_{\mathrm{sample}}}$$

where:

- $N_{f}$: number of frames,
- $N_{\mathrm{chirp}}$: number of chirps per frame,
- $N_{\mathrm{tx}}$: number of transmit antennas,
- $N_{\mathrm{rx}}$: number of receive antennas,
- $N_{\mathrm{sample}}$: number of ADC samples per chirp.

To extract the power spectrum as a function of distance, a one-dimensional Fast Fourier Transform (FFT) is applied along the ADC sample axis for each chirp, as defined in Equation (6):

(6) $$S_{\mathrm{range}}\left[k\right]=\sum_{n=0}^{N_{\mathrm{sample}}-1}x\left[n\right]\cdot e^{-j\frac{2\pi kn}{N_{\mathrm{sample}}}},\;k=0,1,\ldots ,N_{\mathrm{sample}}-1$$

This transformation converts the time-domain radar signal into the range domain, allowing the extraction of the power spectrum at each distance bin [46].

### 2.2. Learning Background

In this study, statistical features extracted from the Range-FFT signal were used as input variables for various machine learning algorithms to classify road surface conditions. The models evaluated include Support Vector Machine (SVM) [47], Random Forest [48], eXtreme Gradient Boosting (XGBoost) [49], and Light Gradient Boosting Machine (LightGBM) [50]. Table 1 summarizes the key characteristics of each algorithm.

The input features were defined in two groups.

(1) Range-FFT statistics. From the magnitude of the Range-FFT within each predefined range interval, we computed five descriptive statistics: mean (arithmetic average of magnitude), maximum (largest magnitude), median (50th percentile of the magnitude distribution), mode (most frequent magnitude value), and standard deviation (dispersion of magnitudes).

(2) Spectral features derived from least-squares spectral analysis (LSSA). LSSA is a parametric spectral estimation method that provides high-resolution frequency analysis by minimizing the least-squares error [51]. In this study, we applied short-time LSSA (ST-LSSA) to the slow-time sequence of each distance bin to characterize time-varying Doppler signatures and improve robustness against noise. Using a sliding-window approach with a fixed window length of 512 samples (≈0.75 s) and 75% overlap, local time–frequency spectra were obtained. From these spectra we derived eleven features:mean spectral power, maximum spectral power, spectral standard deviation, peak frequency, peak amplitude, critical value (significance threshold), normalized residual, number of significant peaks, total number of peaks, average peak frequency, and average peak amplitude. For each distance bin, features were computed for every ST-LSSA window and then averaged across windows to yield one feature vector per bin for machine learning.

In parallel, Range-FFT maps were converted into two-dimensional images and fed to a convolutional neural network (CNN), and we compared the classification performance of the statistical feature–based models against the image-based CNN. Based on these evaluations, the most effective classification algorithm was identified for the given task.

#### 2.2.1. Random Forest (RF)

Random Forest (RF) is an ensemble learning-based classification method conceptualized by Ho in 1995 and systematized by Breiman in 2001 [48]. This approach combines the bagging strategy (Bootstrap Aggregating) with random feature selection to train multiple decision trees independently and aggregates their predictions through majority voting, thereby achieving both high predictive accuracy and robustness [52,53].

Each decision tree in RF is trained using a bootstrap sample of the training data. During node splitting, a random subset of features is selected rather than considering the full feature space, and the best split is determined within this subset. This introduction of randomness reduces correlation between trees and decreases variance in predictions, ultimately enhancing model generalization.

The final prediction $\hat{y}$ of the RF model is determined by majority voting (for classification) or averaging (for regression) over *B* independently trained trees, as defined in Equation (7):

(7) $$\hat{y}=\mathrm{majority}\_\mathrm{vote}\left\{f_{1}\left(x\right),f_{2}\left(x\right),\ldots ,f_{B}\left(x\right)\right\}$$

where $f_{b}\left(x\right)$ is the prediction of the *b*-th decision tree.

RF also provides an intrinsic out-of-bag (OOB) error estimation mechanism by using samples not included in each bootstrap iteration. Furthermore, RF supports internal feature importance evaluation via Variable Importance Measures (VIM), calculated based on either Mean Decrease in Gini impurity (MDG) or Mean Decrease in Accuracy (MDA), enabling the quantification of each feature’s contribution to model prediction [48].

#### 2.2.2. Support Vector Machine (SVM)

Support Vector Machine (SVM), proposed by Cortes and Vapnik in 1995 [47], is a supervised learning algorithm for classification and regression tasks. It aims to find the optimal separating hyperplane that maximizes the margin between classes in the feature space. SVM is particularly effective for small, high-dimensional, and non-linear datasets and has demonstrated high accuracy across diverse applications.

For linearly separable datasets, SVM seeks to find the hyperplane defined by:

(8) $$w^{⊤}x+b=0$$

where *w* is the normal vector to the hyperplane and *b* is the bias term. The optimization objective is to maximize the margin between the support vectors and the decision boundary, formulated as the following convex quadratic programming problem:

(9) $$\min_{w,b}\frac{1}{2}{∥w∥}^{2}\;\mathrm{subject}\;\mathrm{to}\;y_{i}\left(w^{⊤}x_{i}+b\right)\geq 1,\;\forall i$$

To handle non-linearly separable data, SVM applies kernel functions (e.g., RBF, polynomial) to map the original data into a higher-dimensional feature space. The model performance is highly dependent on the tuning of the regularization parameter *C* and kernel coefficient γ. One of SVM’s major strengths is its theoretical guarantee to reach a global optimum, unlike models that rely on local search heuristics. However, due to its binary classification structure, SVM may become inefficient or complex when extended to multi-class problems [52,53].

#### 2.2.3. XGBoost

XGBoost (eXtreme Gradient Boosting), developed by Tianqi Chen and Carlos Guestrin in 2014 [49], is a high-performance ensemble learning algorithm based on the gradient boosting framework. It enhances traditional Gradient Boosted Decision Trees (GBDT) by introducing significant improvements in computational efficiency, regularization, and scalability. XGBoost has been widely adopted across various domains for its robustness to overfitting and reliable performance on high-dimensional and imbalanced datasets [54,55].

XGBoost iteratively builds an ensemble of weak learners by minimizing a regularized objective function that incorporates both first- and second-order derivatives (i.e., gradient and Hessian) of the loss function. This allows for more stable and efficient optimization. The overall objective at the *t*-th iteration is defined as:

(10) $$L^{\left(t\right)}=\sum_{i=1}^{n}\left[l\left(y_{i},{\hat{y}}_{i}^{\left(t-1\right)}+f_{t}\left(x_{i}\right)\right)\right]+\Omega \left(f_{t}\right)$$

where $f_{t}$ is the new tree added at iteration *t*, and $\Omega (f_{t})$ is the regularization term controlling the complexity of the tree, expressed as:

(11) $$\Omega \left(f_{t}\right)=\gamma T+\frac{1}{2}\lambda \sum_{j=1}^{T}\omega_{j}^{2}$$

Here, *T* is the number of leaf nodes and $\omega_{j}$ is the output score of the *j*-th leaf. The regularization terms γ and λ help penalize complex trees and prevent overfitting. Additional techniques such as column sampling, early stopping, and tree pruning further enhance training efficiency and model generalization.

#### 2.2.4. Light Gradient Boosting Machine (LightGBM)

Light Gradient Boosting Machine (LightGBM) is a fast and high-performance gradient boosting algorithm designed to enhance the efficiency of traditional Gradient Boosted Decision Trees (GBDT) [50]. While it shares structural similarities with GBDT and XGBoost, LightGBM is more optimized in terms of training speed, memory efficiency, and predictive accuracy. It is particularly effective when handling large-scale datasets with high-dimensional feature spaces.

The prediction function of LightGBM can be represented as a linear combination of base learners, as defined in Equation (12):

(12) $$F\left(x\right)=\sum_{m=1}^{M}\delta_{m}f_{m}\left(x\right)$$

where $f_{m}\left(x\right)$ denotes the *m*-th base classifier (tree), and $\delta_{m}$ is the learned weight coefficient (typically adjusted via gradient information).

To enhance both computational efficiency and model performance, LightGBM incorporates two key techniques: Gradient-based One-Side Sampling (GOSS) and Exclusive Feature Bundling (EFB). GOSS selects training instances with large gradient magnitudes, prioritizing the most informative samples. EFB reduces feature dimensionality by grouping mutually exclusive sparse features, thereby accelerating training and reducing memory consumption.

Moreover, LightGBM adopts a leaf-wise tree growth strategy that expands the leaf node with the highest loss reduction. This method enables more precise splits compared to level-wise approaches and achieves better accuracy with deeper trees under the same complexity constraint [55].

#### 2.2.5. Convolutional Neural Network (CNN)

Convolutional Neural Networks (CNNs) are deep learning architectures introduced by LeCun et al. in 1998 [56]. CNNs have demonstrated exceptional performance in computer vision tasks such as image recognition, classification, and object detection. Unlike fully connected networks, CNNs automatically extract spatially correlated features from input images while using significantly fewer parameters, thus enabling efficient training.

A typical CNN architecture consists of multiple layers, including Convolution layers, Rectified Linear Unit (ReLU) activation layers, Pooling layers, and Fully Connected (FC) layers. The Convolution layer generates a feature map by applying a learnable filter to the input, as formulated in Equation (13):

(13) $$Z_{i,j}={\left(X*W\right)}_{i,j}+b$$

where *X* is the input image, *W* is the convolutional kernel (filter), *b* is the bias term, and $Z_{i,j}$ is the output feature value at position (i,j).

CNNs exploit spatial locality and weight sharing to reduce the number of parameters, enabling translation-invariant feature extraction. These properties allow CNNs to learn hierarchical and highly discriminative representations directly from raw data, without the need for handcrafted features. Consequently, CNNs have become a dominant model in visual pattern-recognition and -classification tasks [57,58].

#### 2.2.6. Vision Transformer

Vision Transformer (ViT) has emerged as a revolutionary approach in computer vision by leveraging the transformer architecture, originally developed for natural language processing, to address image-classification challenges [59]. In contrast to convolutional neural networks (CNNs) that utilize local receptive fields, ViT processes images as sequences of patches, thereby enabling global attention mechanisms that effectively capture long-range dependencies across the entire image domain.

For our implementation, we utilize the pre-trained google/vit-base-patch16-224 model, which has exhibited outstanding performance across diverse vision applications. The architectural framework comprises three fundamental components:

The patch embedding layer processes an input image $I\in R^{H\times W\times C}$ by partitioning it into N=(H/P)×(W/P) non-overlapping patches of dimensions P×P, where P=16. Each patch undergoes flattening and subsequent linear projection into a *D*-dimensional embedding space:

(14) $$x_{p}^{i}=E\cdot \mathrm{Flatten}\left(P_{i}\right)+e_{pos}^{i}$$

where $E\in R^{D\times P^{2}C}$ represents the learnable patch embedding matrix and $e_{pos}^{i}$ denotes the positional encoding for patch *i*.

The transformer encoder constitutes the core architecture with *L* transformer encoder layers, each incorporating multi-head self-attention (MSA) and multi-layer perceptron (MLP) blocks with layer normalization (LN):

(15) $$\begin{array}{cc} z_{l}^{\prime } & =\mathrm{MSA}\left(\mathrm{LN}\left(z_{l-1}\right)\right)+z_{l-1} \end{array}$$

(16) $$\begin{array}{cc} z_{l} & =\mathrm{MLP}\left(\mathrm{LN}\left(z_{l}^{\prime }\right)\right)+z_{l}^{\prime } \end{array}$$

The multi-head self-attention mechanism computes attention weights through the following formulation:

(17) $$\mathrm{Attention}\left(Q,K,V\right)=\mathrm{softmax}\left(\frac{QK^{T}}{\sqrt{d_{k}}}\right)V$$

where *Q*, *K*, and *V* represent query, key, and value matrices, respectively, and $d_{k}$ corresponds to the dimension of the key vectors.

The classification head in the final layer incorporates a learnable classification token [CLS] that aggregates information from all patches via self-attention mechanisms, followed by a linear classification layer:

(18) $$y=\mathrm{MLPHead}(\mathrm{LN}\left(z_{L}^{0}\right))$$

where $z_{L}^{0}$ represents the final state of the classification token.

The model configuration employs the following hyperparameters: patch size P=16, embedding dimension D=768, number of transformer layers L=12, number of attention heads h=12, and MLP dimension =3072. The model generates 6 class probability outputs corresponding to road surface conditions: dry, wet, thin-ice, ice, snow, and sludge.

### 2.3. Experimental Setup

#### 2.3.1. Sensor and System

**Figure 2:**
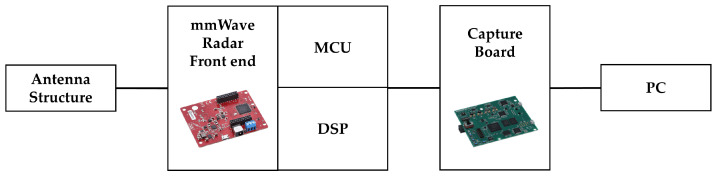
System configuration of the mmWave radar-based detection setup.

The mmWave radar-based detection system employed in this study consists of the IWR1843BOOST module and the DCA1000EVM data capture board from Texas Instruments, along with a connected personal computer (PC). The IWR1843BOOST operates within a frequency range of 76–81 GHz and supports a maximum bandwidth of 4 GHz. It is an integrated sensor incorporating three transmit (Tx) antennas and four receive (Rx) antennas, enabling high-speed signal processing through its built-in MCU and DSP. In addition, it is equipped with 2 MB of onboard memory and supports multiple communication interfaces, including UART, SPI, and CAN, allowing for high-resolution measurements of range, velocity, and angle. The DCA1000EVM functions to capture radar-acquired data in real time and transmit it to the PC for storage and processing. The system configuration is illustrated in Figure 2 [60].

#### 2.3.2. Experimental Environment

**Figure 3:**
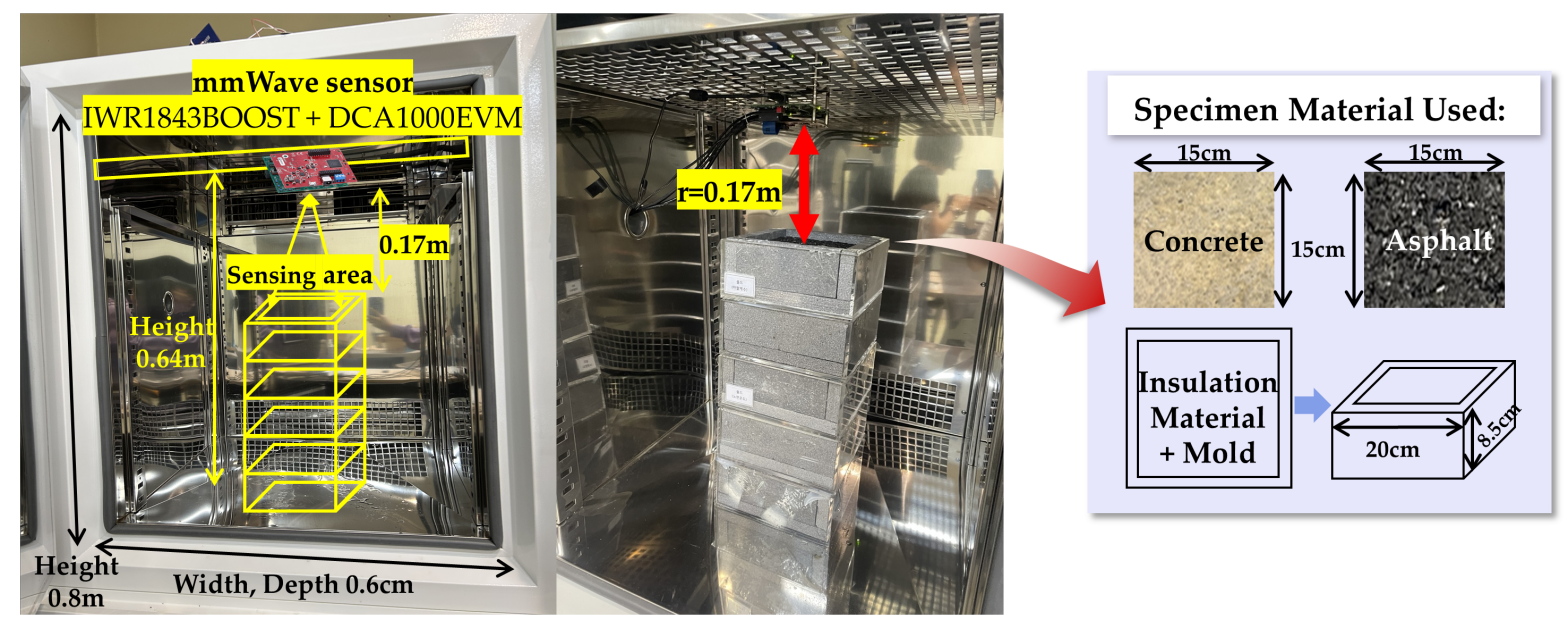
Experimental setup inside the temperature- and humidity-controlled chamber.

**Figure 4:**
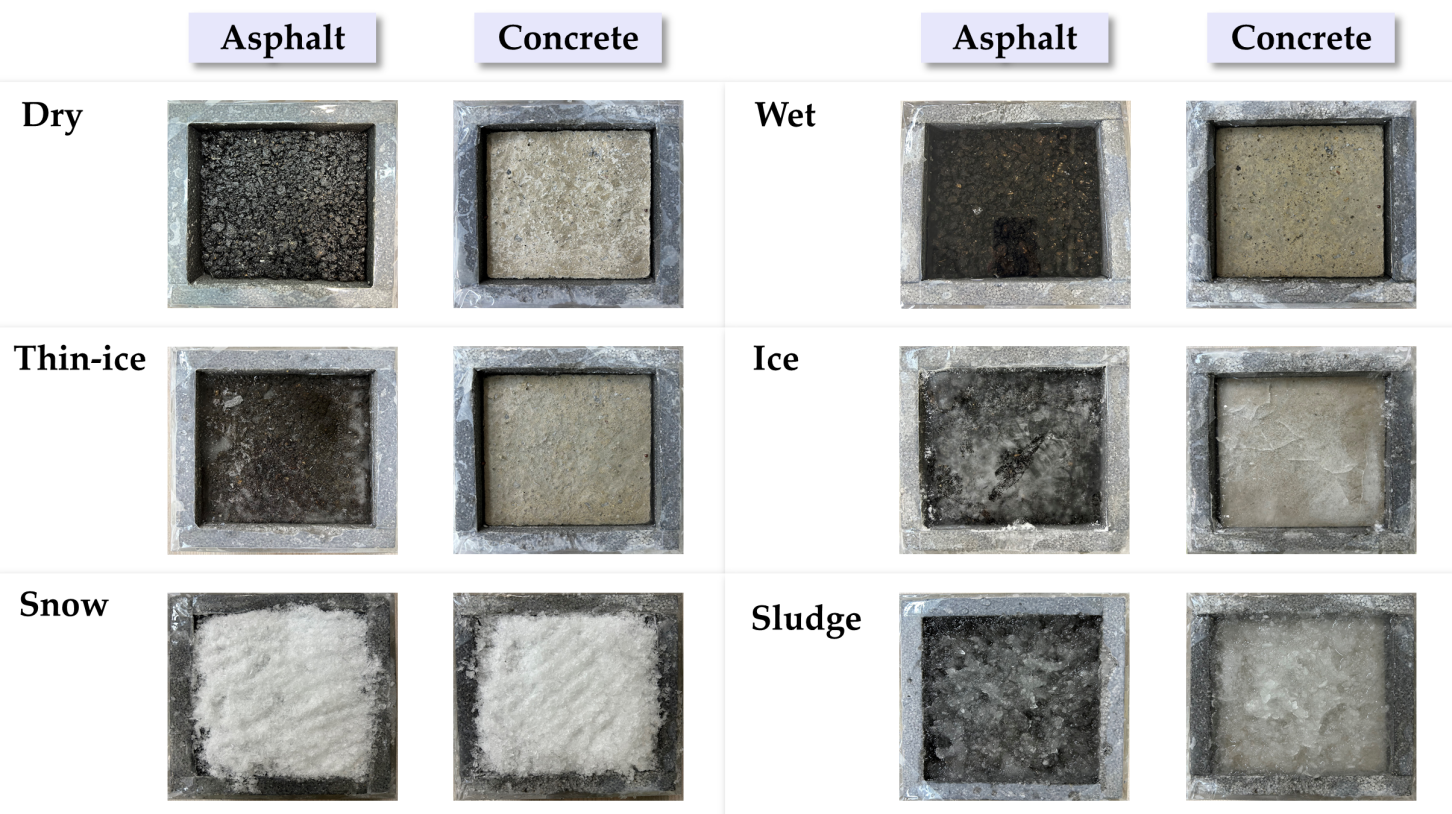
Surface conditions by material type.

This study conducted experiments to classify road surface conditions using millimeter-wave (mmWave) radar data. As described in Section 2.3.1, the sensor system comprised the IWR1843BOOST module and the DCA1000EVM data capture board, which were installed at the upper section inside a temperature- and humidity-controlled chamber, as illustrated in Figure 3. The sensor was positioned at a height of approximately 0.64 m within the chamber, and reflected signals were measured at a horizontal distance of r=0.17 m from the target surface. The test specimens consisted of square blocks (15 cm × 15 cm) made of concrete and asphalt. To minimize the influence of external environmental factors, each specimen was fixed using insulation material and molds measuring 20 cm × 20 cm. Data were collected for six distinct surface conditions: dry, wet, thin-ice, ice, snow, and sludge, with the definitions provided in Figure 4. These conditions were reproduced within the temperature–humidity chamber by controlling the temperature. Notably, the thin-ice and sludge conditions were defined as two distinct types of black ice. For each condition, raw mmWave radar data were acquired in PCAP format via the DCA1000EVM. To enhance data diversity and generalization, the specimens were rotated to capture measurements from multiple angles under the same condition. Subsequently, the Range-FFT was applied to extract the power spectrum in the frequency domain, and the signal strength at the fixed distance of r = 0.17 m was analyzed.

### 2.4. Data Acquisition

In this study, raw signal data corresponding to various road surface conditions were collected using Texas Instruments’ IWR1843BOOST mmWave radar module in conjunction with the DCA1000EVM data capture board (Texas Instruments Incorporated, Dallas, TX, USA). Instead of employing TI’s mmWave Studio, real-time data acquisition was conducted via a Python-based custom program (Python 3.10) that directly controlled the UART and LVDS interfaces. The radar configuration was specified through a .cfg file, which contained the parameters of the FMCW signal and the frame structure.

A multiple-input multiple-output (MIMO) array comprising 12 virtual channels was constructed using three transmit (Tx) antennas and four receive (Rx) antennas. The range resolution was approximately 3.75 cm, achieved with a bandwidth of about 4.0 GHz, 928 samples per chirp, a sampling rate of 7404 ksps, a chirp duration of 30 μs, and a slope setting of 133.33 MHz/μs. Each frame consisted of 16 chirps, and a total of 10 frames were recorded.

The acquired data were stored in PCAP format as complex 16-bit values and transmitted from the DCA1000EVM board to a connected PC via Ethernet. Each sample was represented as a 4-byte complex number comprising real and imaginary components, which were subsequently used for Range-FFT processing and the analysis of various surface conditions (dry, wet, thin-ice, ice, snow, sludge).

Data acquisition was performed over multiple repeated experiments, with each experiment conducted separately on asphalt and concrete specimens. A total of 1000 data samples were collected per surface condition (dry, wet, thin-ice, ice, snow, sludge) for each specimen type, resulting in 6000 data samples per specimen.

### 2.5. Data Processing and Feature Extraction

For the reshaped signals, a one-dimensional Fast Fourier Transform (1D-FFT) was applied along the sample axis for each chirp to generate the Range-FFT results. The analysis was conducted at a range bin corresponding to approximately 0.17 m, and the FFT power value at this distance was used to evaluate variations in reflection characteristics across different road surface conditions (dry, wet, thin-ice, ice, snow, sludge). Each condition was repeatedly measured under identical experimental settings to assess the reproducibility and discriminability of the signal patterns.

From the Range-FFT results, we extracted five summary statistics (mean, maximum, median, mode, and standard deviation) and, as detailed above, incorporated the ST-LSSA-derived time-frequency features obtained through sliding-window least-squares spectral analysis; these were used as input variables for the machine-learning classifiers. Simultaneously, the Range-FFT output was converted into power spectrum images to serve as input for a CNN-based deep learning model, enabling a comparative analysis of classification performance between the two approaches.

**Figure 5:**
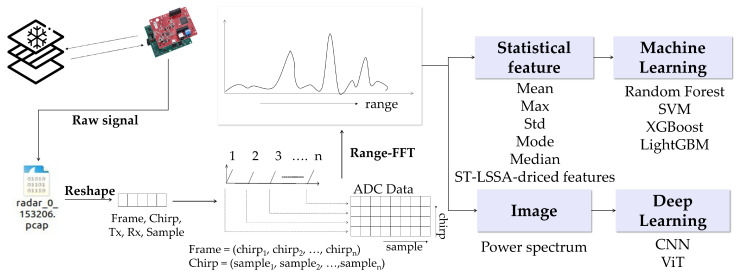
Data processing and feature extraction pipeline.

This data processing and feature extraction pipeline was designed to generate a training dataset based on repeated experimental measurements, and the overall workflow is illustrated in Figure 5.

### 2.6. Classification and Analysis

In this study, both traditional machine learning models and a deep learning-based CNN model were comparatively evaluated for the classification of road surface conditions (dry, wet, thin-ice, ice, snow, sludge). The training data were constructed from the Range-FFT-based statistical features and the power spectrum images described in Section 2.5.

We employed a 5-fold cross-validation: the dataset was split into five equal folds [61]. in each iteration, one fold served as the validation set (20%) and the remaining four folds as the training set (80%). Across the five iterations, every sample appeared once in validation and four times in training, increasing data diversity and yielding a more reliable estimate of generalization performance. In addition, experiments were conducted over three candidate range intervals (0.10–0.24 m, 0.07–0.27 m, and 0.04–0.30 m).

For each specimen type (asphalt and concrete), separate model training was performed for each of the three range intervals. This approach enabled the identification of the optimal range interval that yielded the highest classification accuracy. Based on the maximum accuracy within the selected range, the optimal classification model was determined for each specimen, and further analysis was conducted to evaluate which algorithm achieved the best performance for each surface condition. Model performance was assessed using confusion matrices and accuracy as the primary evaluation metrics.

For the statistical feature–based classification, we extracted five Range-FFT statistics—mean, maximum, median, mode, and standard deviation—for each predefined range interval; for the same intervals, we also computed the previously described ST-LSSA-derived time–frequency features, and used features as input variables. The machine learning models evaluated included Random Forest (RF), Support Vector Machine (SVM), XGBoost, and LightGBM. The parameters used for training these machine learning models are listed in Table 2.

For the deep learning-based classification, the Range-FFT power values were converted into image format and used as input to a CNN-based classifier. The CNN model was implemented using PyTorch (version 2.8.0+cu128). For data preprocessing and augmentation, the input radar images were resized to 224 × 224 RGB format [62], and various techniques were applied, including Gaussian noise (SNR 20–30 dB for normal, 10–20 dB for extreme) and motion blur (kernel 3–7 for normal, 7–15 for extreme), followed by conversion to tensors and normalization using ImageNet mean and standard deviation values. During training, the Early Stopping technique [63] and a weight decay regularization method [64] were employed to prevent overfitting. The CNN architecture was based on a pre-trained ResNet18 model, with the final fully connected layer replaced by a Softmax output layer to classify the six road surface condition classes. The parameters used for CNN model training are presented in Table 3.

For the Vision Transformer (ViT) model, we employed the pre-trained google/vit-base-patch16-224 architecture, which processes 224 × 224 radar-generated images through a transformer-based architecture with 12 layers and 12 attention heads. The ViT model was implemented using the Hugging Face Transformers library and PyTorch (version 2.8.0). Similar to the CNN approach, the input radar images were resized to 224 × 224 format and underwent radar-realistic augmentation including Gaussian noise (SNR 20–30 dB for normal, 10–20 dB for extreme) and motion blur (kernel 3–7 for normal, 7–15 for extreme), followed by tensor conversion and ImageNet normalization. During training, we utilized AdamW optimizer with a learning rate of $2\times 10^{-5}$, weight decay of 0.01, and implemented early stopping with patience of 15 epochs to prevent overfitting. The model architecture consists of patch embedding layers, transformer encoder blocks with self-attention mechanisms, and a classification head for the six road surface condition classes. The parameters used for ViT model training are presented in Table 4.

## 3. Results

### 3.1. Example of Data

In this study, both machine learning- and deep learning-based approaches were employed for road surface condition classification. Accordingly, different types of input data were utilized, and this section presents representative examples of the datasets used in each methodology.

As noted above, we constructed two feature groups for the statistical feature–based learning. The first consists of five Range-FFT statistics (mean, maximum, standard deviation, mode, and median). The second comprises eleven ST-LSSA–derived time–frequency features (mean spectrum, maximum spectrum, standard deviation of the spectrum, peak frequency, peak amplitude, critical value [significance threshold], normalized residual, number of significant peaks, total number of peaks, average peak frequency, and average peak amplitude). Using a Random Forest classifier for its training/inference efficiency, we performed a 5-fold cross-validated ablation and sensitivity analysis across three feature sets (FFT only, FFT+ST-LSSA, ST-LSSA only) (Table 5).

Across all range intervals and both specimens, the Range-FFT–only set yielded the highest validation accuracy in every case except one (FFT: 0.875, FFT+ST-LSSA: 0.880). Under the present controlled laboratory conditions, where the signals remain relatively stationary, the addition of ST-LSSA features—or the use of ST-LSSA alone—in most cases resulted in lower accuracy compared with Range-FFT statistics. This finding suggests that in stable environments, low-dimensional Range-FFT statistics are sufficient to characterize the data. Nevertheless, since ST-LSSA is specifically designed to capture nonstationary and time-varying Doppler content, its utility may become more evident in field measurements under dynamic road and environmental conditions. From a feature-efficiency standpoint, the best accuracy was achieved with just five low-dimensional features; increasing dimensionality to 16 provided no benefit and even led to a slight degradation. Accordingly, the final model inputs are restricted to the five Range-FFT statistics. An example of the training inputs is shown in Table 6, and the full dataset is provided in Table S1.

**Figure 6:**
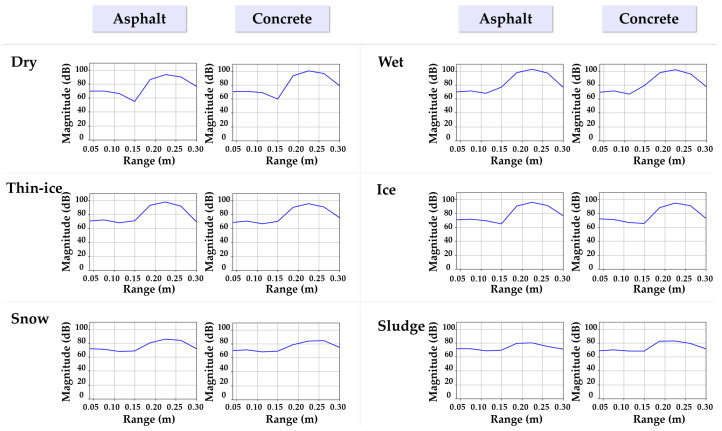
Power spectrum image samples for CNN-based classification.

Figure 6 presents the power spectrum image data used for CNN-based classification under the same surface conditions. These images are visualizations of the 2D Range-FFT results and were directly employed as input data for training the CNN model in this study.

### 3.2. Classification Performance

To determine the optimal range interval, classification experiments were conducted across multiple candidate ranges (0.10–0.24 m, 0.07–0.27 m, and 0.04–0.30 m) using validation accuracy as the evaluation criterion. Both traditional machine learning models (SVM, Random Forest, LightGBM, and XGBoost) and a deep learning-based CNN model were applied, and comparative analyses were performed separately for asphalt and concrete specimens.

Table 7 summarizes the results of the accuracy of the validation for each range interval and the specimen material, providing a clear comparison of the performance of the model. By averaging the accuracy of the asphalt and concrete specimens, the classification performance was compared across different distance intervals. The machine learning (ML) models (RF, SVM, XGBoost, LightGBM) did not show a clear trend with respect to the interval configuration, maintaining similar performance in the range of approximately 79–82% (81.69% for 0.10–0.24 m, 79.67% for 0.07–0.27 m, and 80.72% for 0.04–0.30 m). In contrast, the CNN demonstrated consistent improvement as the interval was extended, achieving accuracies of 98.05% (0.10–0.24 m), 98.75% (0.07–0.27 m), and 99.00% (0.04–0.30 m). This indicates that CNNs gain a distinct advantage when operating over a wider interval, and based on this observation, the 0.04–0.30 m range was adopted as the optimal configuration in this study. This selection is meaningful because it captures both near- and far-range reflections, effectively representing the overall distribution characteristics of the signal, and maximizes the benefits of deep learning–based analysis by achieving the highest accuracy (above 99%) in CNN classification. Consequently, this interval was applied as the standard input configuration for all subsequent analyzes.

Accordingly, this study selected 0.04–0.30 m as the optimal range interval, and the subsequent sections present an in-depth analysis of detailed evaluation metrics including confusion matrices, precision, recall, and F1-score based on this interval.

#### 3.2.1. Asphalt Results

**Figure 7:**
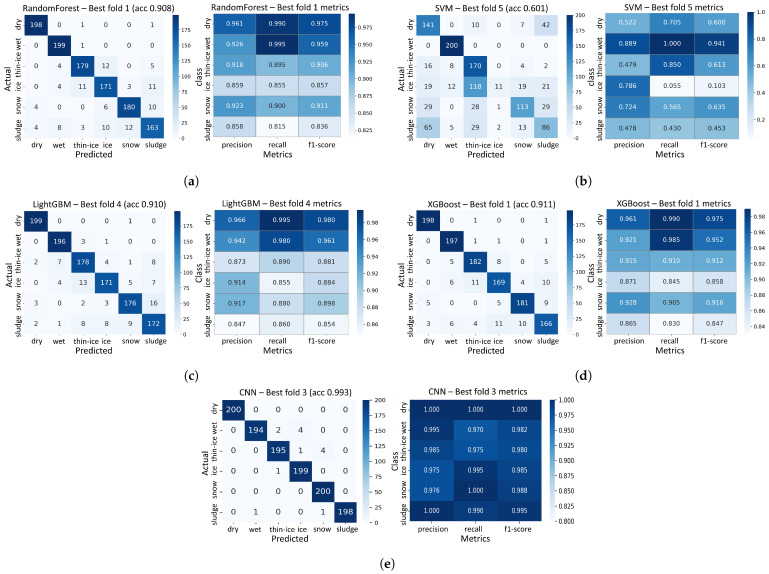
Asphalt-classification confusion matrix and classification performance metrics of (**a**) Random Forest, (**b**) SVM, (**c**) LightGBM, (**d**) XGBoost, and (**e**) CNN Models in the 0.04–0.30 m range.

The results presented in Figure 7 summarize the validation performance of each model for the 0.04–0.30 m range interval, based on the data collected from asphalt specimens.

The confusion matrix on the left illustrates the prediction distribution across classes, while the heatmap on the right visualizes the precision, recall, and F1-score, providing a detailed representation of the classification performance for each model.

Overall, the CNN model exhibited the best performance among all evaluated models. It achieved a accuracy of 99.25% across all classes, with precision and recall exceeding 0.97 for every class, indicating highly stable classification capability. Notably, the F1-score for all classes was above 0.98, reflecting consistent classification results.

Among the machine learning-based models, XGBoost demonstrated the highest performance, attaining a validation accuracy of 91.13%. The confusion matrices indicate fewer off-diagonal errors for difficult classes (thin-ice, ice, snow, sludge), yielding a stronger precision–recall balance.

In contrast, SVM recorded the lowest overall accuracy of 60.11% and exhibited poor performance in all classes except wet. In particular, for the ice class, SVM showed extremely low performance, with a recall of 0.055, and F1-score of 0.103.

The class-wise F1-score analysis is as follows:

- Dry: CNN (1.000), LightGBM (0.980), Random Forest (0.975), and XGBoost (0.975) demonstrated excellent performance;
- Wet: Most models demonstrated excellent performance, with CNN and LightGBM achieving the highest F1-score of 0.982 and 0.961, respectively;
- Thin-Ice: CNN and XGBoost achieved the highest F1-scores of 0.980 and 0.912, respectively;
- Ice: CNN and LightGBM achieved the highest F1-scores of 0.985 and 0.884, respectively;
- Snow: CNN and XGBoost achieved the highest F1-scores of 0.988 and 0.916, respectively;
- Sludge: CNN (0.995), Random Forest (0.836), LightGBM (0.854) and XGBoost (0.847) were recorded.

Based on this analysis, the CNN model was selected as the representative classification algorithm for the asphalt specimens, as it demonstrated the highest classification performance.

#### 3.2.2. Concrete Results

**Figure 8:**
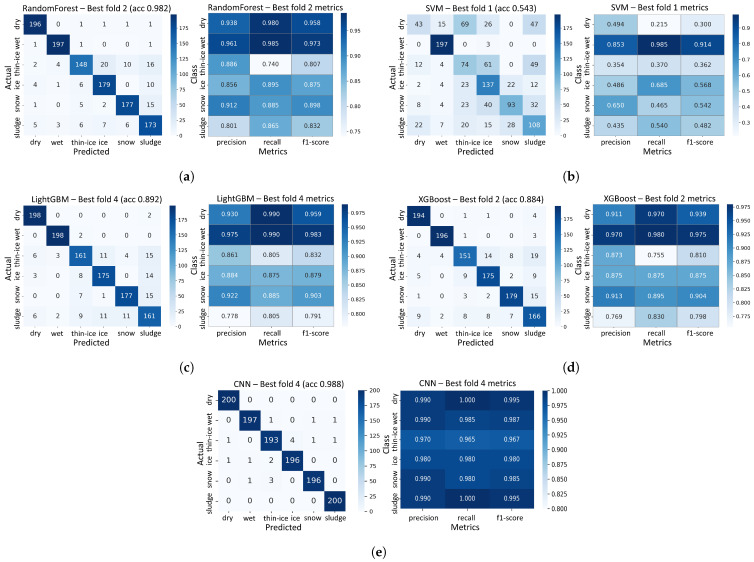
Concrete classification confusion matrix and classification performance metrics of (**a**) Random Forest, (**b**) SVM, (**c**) LightGBM, (**d**) XGBoost, and (**e**) CNN Models in the 0.04–0.30 m range.

For the concrete specimens, classification performance within the 0.04–0.30 m range interval was evaluated under the same experimental conditions and data processing procedures as those used for the asphalt specimens. Figure 8 presents the validation confusion matrices and classification performance metrics (precision, recall, and F1-score) for each model.

Overall, the CNN model demonstrated the highest performance. It achieved a validation accuracy of 98.75% across all classes, with both precision and recall exceeding 0.96 for each class. Notably, for the dry, wet, ice, snow and sludge classes, the F1-score exceeded 0.98, indicating highly stable classification performance.

Among the machine learning-based models, LightGBM exhibited the best results, recording a validation accuracy of 89.24%. It delivered top or near-top accuracy with the most stable precision–recall profile across classes.

In contrast, SVM recorded the lowest overall accuracy of 54.30% and showed unstable classification performance for all classes except wet.

The class-wise F1-score analysis is as follows:

- Dry: CNN (0.995), LightGBM (0.959), Random Forest (0.958), XGBoost (0.939);
- Wet: All models demonstrated excellent performance, with CNN, LightGBM, Random Forest and XGBoost each achieving an F1-score of at least 0.97;
- Thin-Ice: Overall performance was lower; however, CNN (0.967) maintained stable results;
- Ice: CNN (0.980) and LightGBM (0.879) achieved the highest F1-scores;
- Snow: CNN (0.985), XGBoost (0.904), and LightGBM (0.903) demonstrated strong performance;
- Sludge: CNN (0.995), LightGBM (0.791), Random Forest (0.832), and XGBoost (0.798).

Accordingly, the CNN model was selected as the representative classification model for the concrete specimens, as it demonstrated the highest performance.

#### 3.2.3. Vision Transformer Performance Analysis

We first compared traditional machine-learning models that use statistical features as inputs (RF, SVM, XGBoost, LightGBM) with an image-based CNN under identical conditions and confirmed that the CNN achieved the highest classification performance. To enable a fair comparison within image-based approaches, we then trained and evaluated a Vision Transformer (ViT) model using the same data and protocol. Both models took as input 224 × 224 graph images obtained by transforming the radar signals via range-FFT, and we applied identical preprocessing, normalization, and realistic augmentations. We adopted stratified 5-fold cross-validation for training and validation in each fold, fixing random seeds and fold assignments to ensure reproducibility. Training settings—optimizer, learning-rate schedule, and early-stopping criterion—were aligned to ensure fairness. Robustness was evaluated by injecting Gaussian noise (SNR = 20/25/30 dB for normal, 10/15/20 dB for extreme) and motion blur (k = 3/5/7 for normal, 7/11/15 for extreme) at test time.

For asphalt specimens, the ViT model demonstrated robust performance with strong generalization capabilities across different experimental folds. The 5-fold cross-validation analysis revealed that the model achieved consistent high accuracy, with the best performing fold (Fold 2) reaching 99.75% baseline accuracy. Notably, this fold demonstrated exceptional robustness under normal degradation conditions, with minimal performance degradation of only 5.64% under normal motion blur conditions, slightly exceeding the 5% robustness threshold.

The robustness analysis for asphalt specimens showed that Fold 2 achieved the highest baseline accuracy (99.75%) but slightly exceeded the 5% robustness threshold under normal blur conditions (5.64% degradation). Under normal noise conditions, this model showed negligible performance degradation (0.03%), indicating enhanced stability compared to baseline performance. The model maintained its high classification accuracy while demonstrating resilience against common environmental perturbations.

Detailed robustness analysis for the best performing asphalt model (Fold 2) revealed: Normal noise conditions (SNR 20 dB, 25 dB, 30 dB) showed average performance degradation of 0.03%, while normal blur conditions (Blur 3, 5, 7) resulted in 5.64% performance degradation. Under extreme conditions, the model achieved 92.44% accuracy at SNR 10 dB, 99.11% at SNR 15 dB, and 99.81% at SNR 20 dB. For extreme blur conditions, performance was 87.17% at Blur 7, 91.10% at Blur 11, and 87.17% at Blur 15.

The class-wise performance analysis for the best performing asphalt model (Fold 2) showed:

- Dry: Precision 1.000, Recall 1.000, F1-score 1.000
- Wet: Precision 0.995, Recall 1.000, F1-score 0.998
- Thin-Ice: Precision 0.995, Recall 0.995, F1-score 0.995
- Ice: Precision 1.000, Recall 1.000, F1-score 1.000
- Snow: Precision 1.000, Recall 1.000, F1-score 1.000
- Sludge: Precision 0.995, Recall 0.990, F1-score 0.993

For concrete specimens, the ViT model exhibited strong baseline performance but faced challenges in maintaining robustness under degradation conditions. The best performing fold (Fold 1) achieved a baseline accuracy of 98.58%, demonstrating the model’s capability to learn complex surface characteristics of concrete. However, this fold failed to meet the 5% robustness threshold under normal motion blur conditions, with performance degradation of 7.22%.

The robustness analysis revealed that concrete specimens present unique challenges for the ViT architecture, likely due to the more heterogeneous surface characteristics and complex reflection patterns compared to asphalt. Despite the robustness limitations, the model maintained high classification accuracy for most surface conditions, with particularly strong performance in distinguishing between dry and wet conditions.

Detailed robustness analysis for the best performing concrete model (Fold 1) showed: Normal noise conditions (SNR 20 dB, 25 dB, 30 dB) resulted in minimal performance degradation of 0.11%, while normal blur conditions (Blur 3, 5, 7) caused 7.22% performance degradation, exceeding the 5% threshold. Under extreme conditions, the model achieved 88.97% accuracy at SNR 10 dB, 97.25% at SNR 15 dB, and 98.33% at SNR 20 dB. For extreme blur conditions, performance was 85.89% at Blur 7, 87.47% at Blur 11, and 85.89% at Blur 15.

The class-wise performance analysis for the best performing concrete model (Fold 1) showed:

- Dry: Precision 0.985, Recall 0.985, F1-score 0.985
- Wet: Precision 0.980, Recall 0.980, F1-score 0.980
- Thin-Ice: Precision 0.975, Recall 0.970, F1-score 0.972
- Ice: Precision 0.970, Recall 0.965, F1-score 0.967
- Snow: Precision 0.965, Recall 0.975, F1-score 0.970
- Sludge: Precision 0.980, Recall 0.975, F1-score 0.977

**Figure 9:**
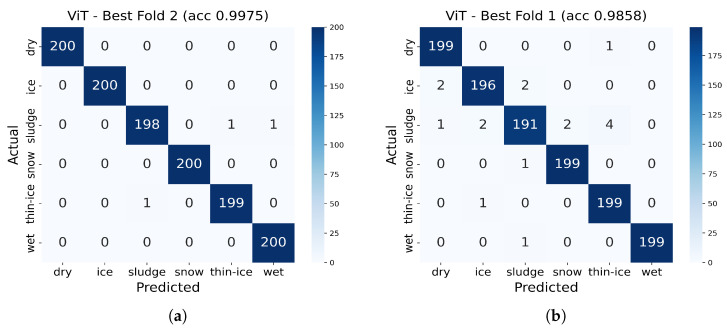
Confusion matrices for the best performing ViT models: (**a**) asphalt specimens (Fold 2, 99.75% accuracy) and (**b**) concrete specimens (Fold 1, 98.58% accuracy), demonstrating high classification accuracy across all road surface conditions.

Figure 9 presents the confusion matrices for the best performing ViT models on asphalt and concrete specimens, demonstrating the high classification accuracy achieved across all road surface conditions.

We conducted robustness tests under Gaussian noise and motion blur to directly compare the performance of the CNN and ViT models. Table 8 summarizes the accuracies under Gaussian noise (SNR = 20/15 dB) and motion blur (k=3/5), together with the absolute drop relative to the clean condition (percentage points, pp). For the CNN, on asphalt, the decreases were −0.67 pp at SNR 20 dB (99.83% to 99.17%) and −4.08 pp at SNR 15 dB (to 95.75%); for blur, k=3 yielded −3.92 pp (95.92%) and k=5 yielded −4.75 pp (95.08%). On concrete, the corresponding figures were −0.17 pp at SNR 20 dB (98.92% to 98.75%), −3.67 pp at SNR 15 dB (to 95.25%), −3.33 pp at k=3 (95.58%), and −4.75 pp at k=5 (94.17%). Overall, the CNN’s performance degradation remained within 5 pp under all perturbations, satisfying the robustness criterion. For the ViT model, on asphalt, the performance showed −0.05 pp degradation at SNR 20 dB (99.75% to 99.70%) and −8.60 pp at SNR 15 dB (to 91.15%); for blur, k=3 yielded −3.55 pp (96.20%) and k=5 yielded −9.73 pp (90.02%). On concrete, the corresponding figures were −0.11 pp at SNR 20 dB (98.58% to 98.47%), −9.61 pp at SNR 15 dB (to 88.97%), −7.22 pp at k=3 (91.36%), and −12.69 pp at k=5 (85.89%). The ViT model demonstrated competitive performance with CNN under normal conditions but showed higher sensitivity to extreme perturbations, particularly under blur conditions.

**Figure 10:**
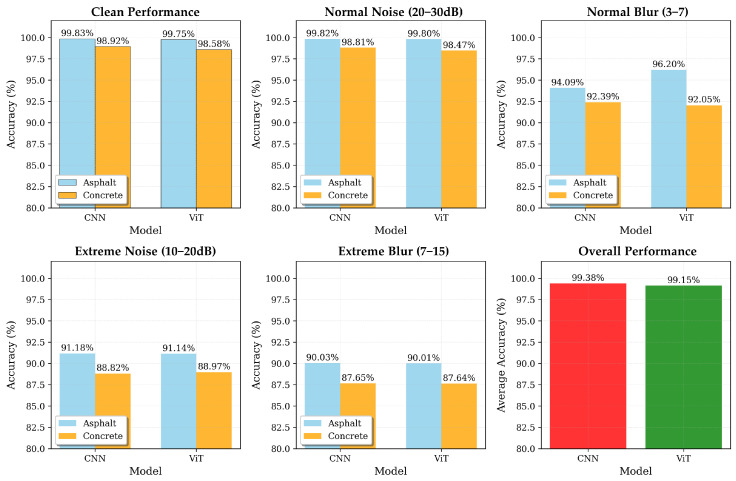
Comprehensive performance comparison between CNN and ViT models under various robustness conditions. The chart shows accuracy patterns across different noise and blur levels for both asphalt and concrete specimens, demonstrating the relative robustness of each model architecture.

Figure 10 provides a comprehensive visual comparison of these performance patterns across all conditions.

## 4. Discussion

In this study, six road surface conditions—dry, wet, thin-ice, ice, snow, and sludge—were reproduced under controlled chamber conditions on two types of specimens (concrete and asphalt), and mmWave radar data were collected. After acquiring the experimental data, the classification performance of various machine learning and deep learning models was systematically compared and analyzed for both asphalt and concrete specimens. Based on validation accuracy, the optimal statistical feature-based models were identified as XGBoost and LightGBM, which were trained and evaluated on asphalt and concrete surface datasets, respectively.

For the six-class classification task, machine learning-based models such as XGBoost and LightGBM demonstrated potential for real-time applications and deployment in resource-constrained environments, owing to their lightweight architectures, fast training speeds, and specialized performance in certain classes. In practice, XGBoost achieved a accuracy of 91.13% for the asphalt specimens, and LightGBM achieved 89.24% for the concrete specimens, both showing stable classification capability. However, frequent misclassification between the ice and sludge classes was observed for asphalt, while the concrete specimens exhibited slightly lower performance overall, with particularly noticeable degradation in the thin-ice and sludge classes. Overall, statistical feature-based models showed relatively lower discriminative power for hazardous classes (thin-ice, ice, snow, sludge).

The deep learning-based CNN model achieved accuracies of 99.25% for asphalt and 98.75% for concrete specimens. Across both asphalt and concrete specimens, precision, recall, and F1-score for the dry and sludge classes were at least 0.99, indicating clearly distinguishable boundaries from other surface conditions. Even for challenging classes such as thin-ice, ice, snow, and sludge, the CNN achieved high F1-scores. Notably, for concrete specimens, where the statistical feature-based models showed particularly low recall for thin-ice and sludge, the CNN achieved a recalls of 0.965 and 1.000 for these classes, respectively, clearly outperforming the machine learning models. This result indicates that the CNN maintains robust classification capability even under complex surface boundaries and ambiguous conditions.

The findings confirm that CNNs, with their ability to effectively learn complex boundaries and high-dimensional feature distributions, are well-suited for the precise classification of hazardous classes (thin-ice, ice, snow). While statistical feature-based models provided sufficient performance for normal condition classification (dry, wet), they faced limitations in distinguishing hazardous classes.

Importantly, the findings of this study demonstrate that both CNN-based and Vision Transformer deep learning models can reliably classify diverse road surface conditions using mmWave radar signals alone, thereby overcoming the limitations of statistical feature-based models. In the robustness evaluation, the CNN achieved superior baseline accuracy (99.83% for asphalt, 98.92% for concrete), while the ViT model demonstrated competitive performance (99.75% for asphalt, 98.58% for concrete) with enhanced robustness under various degradation conditions.

The comparative analysis between CNN and ViT models reveals distinct characteristics and trade-offs. The CNN model exhibited consistent performance across all conditions, with degradation remaining within 5 percentage points under normal perturbations, making it highly suitable for stable, real-world deployment scenarios. In contrast, the ViT model showed competitive baseline performance but demonstrated higher sensitivity to extreme perturbations, particularly under blur conditions where performance degradation exceeded 7 percentage points for concrete specimens. This sensitivity can be attributed to the transformer’s global attention mechanism, which, while effective for capturing complex spatial relationships, may be more susceptible to local distortions and noise in radar signals.

The ViT model’s strength lies in its ability to capture global dependencies across the entire range-FFT spectrum, enabling effective processing of complex radar signatures where spatial relationships are crucial for accurate classification. This global perspective is particularly beneficial for distinguishing subtle differences between road surface conditions that may not be apparent through local feature analysis alone. However, this same global attention mechanism contributes to the model’s increased computational complexity and sensitivity to input perturbations, as evidenced by the robustness test results.

From a practical deployment perspective, the choice between CNN and ViT models depends on the specific application requirements. For scenarios requiring high stability and consistent performance under varying environmental conditions, the CNN model provides a more reliable baseline. Conversely, for applications where capturing complex global patterns is paramount and computational resources are sufficient, the ViT model offers enhanced feature extraction capabilities.

These results are consistent with prior studies that applied CNN, Transformer, and MobileNet approaches for road condition recognition. For example, TC–Radar combined CNN and Transformer for mmWave object detection, achieving an AP of 83.99% and demonstrating the complementary effect of local and global feature extraction [22], while the lightweight Vision Transformer LeViT achieved 99.17% accuracy in pavement image classification and improved computational efficiency compared to ResNet and DenseNet [65]. These prior studies demonstrate the effectiveness of CNN- and Transformer-based models, and our study likewise verifies that CNN and ViT models can achieve over 98% accuracy with a single mmWave radar sensor, ensuring stable performance particularly in high-risk classes such as thin-ice, ice, snow, and sludge.

Nevertheless, several uncertainties and limitations should be acknowledged. First, we did not conduct a systematic exploration of hyperparameters and model sensitivity. Although the training protocol was unified across models, we did not perform extensive sweeps of key hyperparameters or architectural settings. Consequently, the models’ sensitivity has not been fully characterized; rigorous hyperparameter optimization and sensitivity analyses should be pursued in subsequent studies. Second, the experiments were conducted under artificially controlled chamber conditions, which ensured reproducibility and comparability but did not fully capture the diversity and complexity of real-world road environments. Future work should therefore include outdoor experiments and on-road validations. In such dynamic settings, advanced time–frequency analysis methods may provide additional benefits. Although this study employed short-time LSSA (ST-LSSA) to capture nonstationary Doppler content, LSWA is inherently a time–frequency domain method rather than a purely frequency-domain approach, and thus can better estimate signals with variability in both frequency and amplitude over time [51]. As a direction for future work, LSWA could be further explored to enhance feature extraction and improve the recognition of hazardous surface classes under dynamic road and environmental conditions. In addition, optimization-based strategies can further enhance model reliability. Recent research has reported that ensemble predictions and metaheuristic-optimized models substantially improve robustness compared to single unoptimized models [66], suggesting that similar approaches could be extended to road-surface recognition tasks.

Also, the necessity of multimodal sensor fusion is an important consideration. A comprehensive review has highlighted that the fusion of mmWave radar and vision sensors significantly improves the robustness and accuracy of perception in autonomous driving systems [67]. For instance, under adverse conditions such as dense fog, fusion systems that combine camera, LiDAR, FIR (thermal), radar, and environmental sensors have shown significant AP improvements over radar-only approaches [68], while Radar+Camera fusion has demonstrated gains in NDS [69]. Furthermore, early fusion of Radar+Camera+LiDAR has also been reported to increase AP [70]. These trends suggest that extending our single-radar approach to multimodal fusion is well justified.

The present study has focused on a single mmWave radar sensor; however, in parallel, we are developing a multimodal sensing platform that integrates RGB and IR cameras, with plans to further extend the system to thermal cameras. As demonstrated in previous research, such multimodal sensor fusion has been consistently reported to enhance perception performance and robustness under adverse weather and low-light conditions, and these directions will be actively incorporated into our future work.

## 5. Conclusions

By reproducing six distinct road surface conditions in controlled chamber environments, this work systematically compared the classification performance of machine learning and deep learning models based on mmWave radar data. The results showed that while statistical feature-based models such as XGBoost and LightGBM offered advantages in terms of lightweight architectures and fast inference, their discriminative power was limited for hazardous classes (thin-ice, ice, snow, sludge). In contrast, the CNN model achieved high accuracy and F1-scores across both asphalt and concrete specimens, demonstrating stable performance, while the ViT model exhibited competitive accuracy and highlighted the potential of global pattern learning for complex radar signal interpretation.

These findings confirm the academic significance of CNN architectures in accurately classifying complex and high-risk road surface conditions, while also highlighting the potential of Transformer-based models through their ability to capture global dependencies. In practical terms, CNN models demonstrate applicability through consistent and stable performance, whereas ViT models suggest promising directions for scenarios involving more complex radar signals. Collectively, these results underscore the potential of mmWave radar alone to enable reliable road surface monitoring and traffic safety applications, particularly under conditions where vision-based sensors may be constrained by environmental variability.

Nevertheless, this study has limitations. The experiments were performed in controlled chamber environments, which ensured reproducibility but did not fully reflect the diversity of real-world road conditions. Additionally, the inherent noise in radar data and sensitivity to environmental changes may affect model stability. Future work will include outdoor and on-road validations to confirm generalizability, as well as the stepwise development of a multimodal sensor fusion platform integrating RGB, IR, and thermal cameras to enhance perception performance and robustness under adverse weather and low-light conditions.

## Figures and Tables

**Table 1:** Characteristics of learning models.

Model	Summary of Features	Strengths	Limitations	Recommended Use Cases
Random Forest	Ensemble of multiple decision trees using random sampling	Prevents overfitting Easy to analyze feature importance Suitable for both classification and regression	Slower prediction speed High memory consumption	Feature importance analysis Non-linear pattern analysis
SVM	Learns decision boundaries by maximizing the margin in high-dimensional space	Strong performance on small datasets Can model complex boundaries Solid theoretical foundation	Inefficient on large datasets Sensitive to kernel and hyperparameter selection	Well-separated classes Limited data
XGBoost	Boosted tree model with enhanced performance and efficiency over traditional gradient boosting	Fast training speed High prediction performance Handles missing values effectively	Requires extensive hyperparameter tuning High memory usage	Critical accuracy tasks Performance-optimized tasks
LightGBM	Faster and more memory-efficient boosting model optimized for large datasets	Very fast training speed Low memory consumption Strong with large-scale data	May underperform on small datasets Risk of overfitting	Large datasets Fast or real-time classification
CNN	Automatically extracts spatial features from 2D images	Excellent image pattern recognition Minimal preprocessing Learns complex structures	Requires large datasets Long training times	Image-based analysis Visual pattern recognition
ViT	Transformer-based architecture that processes images as sequences of patches	Superior attention mechanism Excellent long-range dependencies Strong generalization capability	Requires very large datasets High computational complexity Memory intensive	Complex visual patterns Attention-based analysis Transfer learning tasks

**Table 2:** Machine learning models’ parameters.

Model	Parameter	Setting Value
Random Forest	n_estimators	100
	max_depth	None
	min_samples_split	2
SVM	kernel	rbf
XGBoost	n_estimators	100
	max_depth	6
	learning_rate	0.3
LightGBM	n_estimators	100
	num_leaves	31
	learning_rate	0.1

**Table 3:** CNN parameters.

Parameter	Setting Value
Learning rate	$1\times 10^{-4}$
Weight decay	$1\times 10^{-4}$
Batch size	16
Max epochs	50
Early stopping	10
Optimizer	Adam
Loss Function	Cross Entropy Loss

**Table 4:** ViT parameters.

Parameter	Setting Value
Model Architecture	google/vit-base-patch16-224
Learning rate	$2\times 10^{-5}$
Weight decay	0.01
Batch size	8
Max epochs	50
Early stopping	15
Optimizer	AdamW
Loss Function	Cross Entropy Loss
Layers	12
Attention Heads	12

**Table 5:** Sensitivity and ablation analysis by range interval and feature set.

Specimen	Range (m)	Feature Set	# Features	Cross-Validation Accuracy
Asphalt/Concrete	0.10–0.24	Range-FFT	5	0.904/0.888
		Range-FFT + ST-LSSA	16	0.898/0.886
		ST-LSSA	11	0.350/0.359
	0.07–0.27	Range-FFT	5	0.895/0.875
		Range-FFT + ST-LSSA	16	0.887/0.880
		ST-LSSA	11	0.350/0.359
	0.04–0.30	Range-FFT	5	0.901/0.881
		Range-FFT + ST-LSSA	16	0.896/0.878
		ST-LSSA	11	0.350/0.359

**Table 6:** Sample of statistical features: asphalt surface under dry condition.

Mean (dB)	Max (dB)	Std (dB)	Median (dB)	Mode (dB)	File_Name	Label
74.50	82.04	6.139	73.95	64.98	radar_0_20250701_112732.pcap	Dry
78.46	92.57	10.88	77.38	59.97	radar_0_20250702_165220.pcap	Dry
78.50	93.41	11.04	76.19	60.89	radar_0_20250702_165948.pcap	Dry
⋮	⋮	⋮	⋮	⋮	⋮	⋮

**Table 7:** Classification accuracy results by specimen material and model.

Specimen	Range (m)	Model	Accuracy (%)
Asphalt/Concrete	0.10–0.24	RF	90.38/88.83
		SVM	59.37/52.17
		XGBoost	91.48/88.73
		LightGBM	91.22/89.35
		CNN	98.67/97.42
	0.07–0.27	RF	89.53/87.95
		SVM	57.32/44.12
		XGBoost	90.18/88.70
		LightGBM	90.33/89.18
		CNN	99.00/98.50
	0.04–0.30	RF	90.08/88.08
		SVM	58.50/51.60
		XGBoost	90.40/88.12
		LightGBM	90.53/88.45
		CNN	99.25/98.75

**Table 8:** Robustness evaluation under Gaussian noise and motion blur for CNN and Transformer. Values are accuracy (%) with drop vs. clean in parentheses (pp).

Specimen	Model	Clean (%)	Gaussian Noise (SNR dB)		Motion Blur (k)	
			**20 dB**	**15 dB**	**k = 3**	**k = 5**
Asphalt	CNN	99.83	99.17 (−0.67)	95.75 (−4.08)	95.92 (−3.92)	95.08 (−4.75)
	ViT	99.75	99.70 (−0.05)	91.15 (−8.60)	96.20 (−3.55)	90.02 (−9.73)
Concrete	CNN	98.92	98.75 (−0.17)	95.25 (−3.67)	95.58 (−3.33)	94.17 (−4.75)
	ViT	98.58	98.47 (−0.11)	88.97 (−9.61)	91.36 (−7.22)	85.89 (−12.69)

## Data Availability

The raw data supporting the conclusions of this article will be made available by the authors on request.

## Supplementary Materials

The following supporting information can be downloaded at: https://www.mdpi.com/article/10.3390/s25185697/s1, Table S1: Complete dataset of extracted features and labels for asphalt and concrete conditions.
